# A Nanotechnology-Based Platform for Extending the Pharmacokinetic and Binding Properties of Anti-methamphetamine Antibody Fragments

**DOI:** 10.1038/srep12060

**Published:** 2015-07-10

**Authors:** Nisha Nanaware-Kharade, Shraddha Thakkar, Guillermo A. Gonzalez III, Eric C. Peterson

**Affiliations:** 1 Department of Pharmacology and Toxicology, College of Medicine, University of Arkansas for Medical Sciences, 4301 West Markham St., # 611, Little Rock, Arkansas 72205, USA; 2 Department of Physiology and Biophysics, College of Medicine, University of Arkansas for Medical Sciences, 4301 West Markham St., # 750, Little Rock, Arkansas 72205, USA

## Abstract

To address the need for effective medications to aid in the treatment of methamphetamine (METH) abuse, we used a nanotechnology approach to customize the *in vivo* behavior of an anti-METH single chain antibody (scFv7F9Cys). Anti-METH scFv7F9Cys was conjugated to dendrimer nanoparticles via a polyethylene glycol (PEG) linker to generate high-order conjugates termed dendribodies. We found that the high affinity (K_D_ = 6.2 nM) and specificity for METH was unchanged after nanoparticle conjugation. The dendribodies were administered in an i.v. bolus to male Sprague Dawley rats after starting a s.c. infusion of METH. The PCKN values for clearance and volume of distribution of scFv7F9Cys after conjugation to dendrimers decreased 45 and 1.6-fold respectively, and the terminal elimination half-life increased 20-fold. Organ distribution of scFv7F9Cys and dendribody in blood and urine agreed well with the PCKN data. Renal clearance appeared to be the major route of elimination for both experimental medications. We have thus successfully developed a novel multivalent METH-binding nanomedicine by conjugating multiple anti-METH scFvs to dendrimer nanoparticles, extending the scFv half-life from 1.3 (±0.3) to 26 (±2.6) hr. These data suggest that the dendribody design could be a feasible platform for generating multivalent antibodies with customizable PCKN profiles.

METH-specific IgG monoclonal antibodies (mAbs) and antibody fragments such as single chain variable fragments (scFv) are promising new medications being developed to treat methamphetamine (METH) addiction. These therapies act as a pharmacokinetic (PCKN) antagonists by altering the disposition of METH, thus removing and/or preventing METH from reaching its multiple sites of action^1^,^2^,^3^,^4^,^5^. Due to the diverse treatment modalities needed for drug abuse (*e.g.*, short-acting medication for overdose, or long-acting for chronic treatment), the ability to customize PCKN parameters of a single antibody or fragment for multiple indications could be advantageous. We previously described a design strategy for conjugating multiple therapeutic anti-METH scFvs to Generation 3 (G3) polyamidoamine (PAMAM) dendrimer nanoparticles via polyethylene glycol (PEG) crosslinker to generate multivalent G3-PEG-scFv conjugates termed dendribodies^6^. Here we report that the PCKN profile and efficacy of an anti-METH scFv7F9Cys was extended by conjugating it to a dendrimer delivery system. The dendrimer conjugated scFv7F9Cys exhibited prolonged activity *in vivo* due to increased residence time compared to the unconjugated scFv7F9Cys, mainly due to reduced clearance (Cls).

With the rise of antibody fragments and alternative binding scaffolds devoid of Fc binding regions, various strategies to increase the t_1/2_ of these proteins have been developed^7^,^8^. There are two major approaches that have been used to alter the PCKN of scFv molecules. The first is multimerization using recombinant manipulation, however scFvs tend to self-associate in unpredictable mixtures of dimers, trimers, and larger molecular weight complexes leading to production issues and poor reproducibility of the therapeutic properties. The second is chemical conjugation to a PEG chain. This strategy does extend the *in vivo* half-life but does not increase the binding valency of the scFv, nor its potency^9^,^10^. Conjugation to PEG has even been reported to cause a decrease in the affinity of some conjugated antibody fragments^11^,^12^. Here we report a dendribody design that converts single METH binding scFv into a multivalent nanomedicine that in theory can bind multiple METH molecules^13^ while significantly extending the PCKN half-life of the experimental medication.

## Results and Discussion

**Figure 1:**
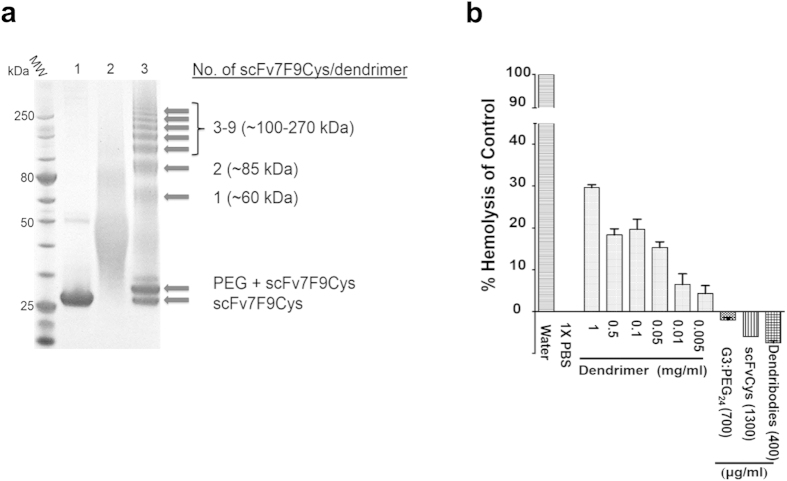
**a** SDS-PAGE reducing gel showing the PEG_24_ modified G3 PAMAM dendrimer to scFv7F9Cys (dendribody) crosslinking reaction: (lane 1) purified scFv7F9Cys, (lane 2) PEG_24_:G3 dendrimer (reaction ratio 11:1), and (lane 3) dendribody conjugation reaction incubated at room temperature and synthesized in conjugation buffer adjusted to pH 6.4. The scFv7F9Cys dendrimer conjugation resulted in higher-order dendribodies with on average, seven scFv7F9Cys molecules conjugated per dendrimer nanoparticle. **b** *In vitro* hemolysis assay to determine the safety of the dendribody medication. G3 PAMAM dendrimers exhibit concentration dependent hemolysis, whereas PEG modified dendrimers, unconjugated proto-type scFv6H4Cys, and dendribodies are non-hemolytic. PEG modified dendrimers, scFv6H4Cys, and dendribodies protected the erythrocytes from hemolysis better than the control buffer (phosphate buffer saline, PBS).

**Figure 2:**
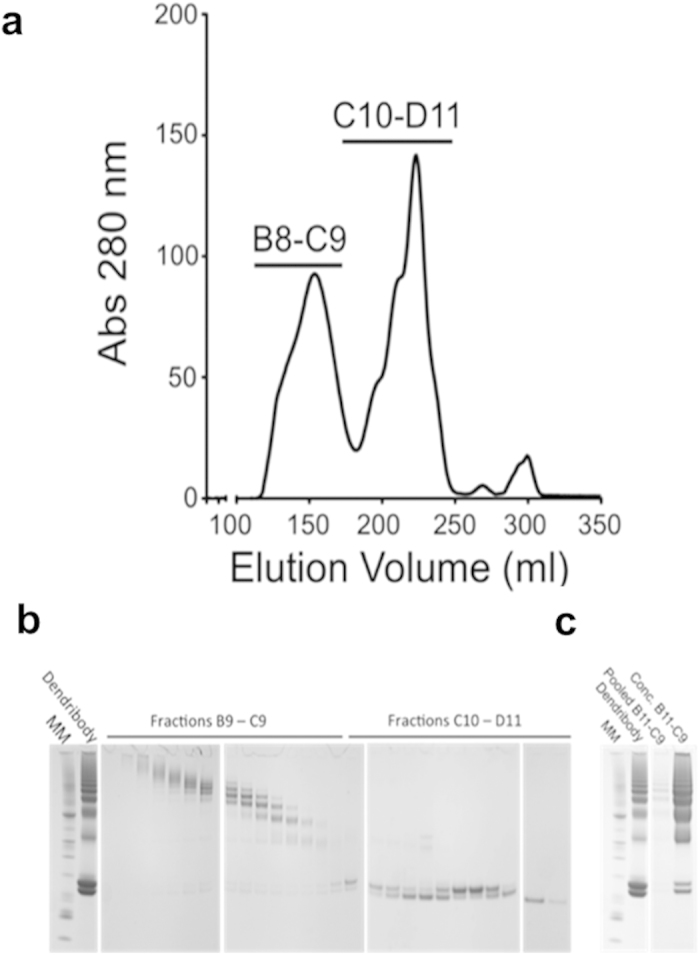
**a** Separation profile of dendribodies from unreacted scFv7F9Cys using size exclusion chromatography. Peak assignments: (B8-C9) dendribodies and (C10-D11) PEG modified and unreacted scFv7F9Cys. **b** SDS-PAGE analysis of the fractions. **c**, SDS-PAGE analysis of pooled and concentrated elution fractions B11-C9.

We previously reported our anti-METH scFv6H4Cys as a prototype antibody fragment to demonstrate the initial synthesis feasibility of the dendribody platform^6^. However, for *in vivo* proof-of-principle evidence of efficacy of the dendribody platform we shifted to another of our high affinity anti-METH scFvs, scFv7F9Cys. This was done for two reasons 1) the chimeric anti-METH Ch-mAb7F9 successfully completed a Phase 1a safety study (NCT01603147), suggesting a clearer route to the clinic for this antibody fragment^14^, and 2) SDS-PAGE analysis showed that scFv7F9Cys also resulted in higher-order dendribodies (increased multivalency) compared to prototype scFv6H4Cys (Fig. 1a)^6^. To prepare the scFv7F9Cys dendribodies for *in vivo* testing, the synthesis reactions were purified by size exclusion chromatography (SEC) to separate the dendribodies from unreacted scFv7F9Cys, PEG modified scFv7F9Cys, and dendrimers (Fig. 2a). Dendribodies with higher numbers of scFv7F9Cys eluted from the column in early fractions followed by lower-order dendribodies. PEG modified and unreacted scFv7F9Cys eluted predominantly in the later fractions (Fig. 2b). All fractions were analyzed by SDS-PAGE. The initial fractions of enriched dendribodies were pooled and concentrated and used for further studies (Fig. 2c).

Previously, it has been reported that dendrimers with exposed cationic surface groups exert hemolytic effects^15^. Hemolysis results from a serious damage to the membrane lipid bilayer and loss of cell integrity. The surface membrane of erythrocytes is negatively charged under physiological conditions. It is hypothesized that through initial adhesion to the cell surface via electrostatic attraction dendrimers cause disruption by creating holes into the cell membrane^16^. However, it is known that PEG modification of PAMAM dendrimers masks the cationic groups resulting in reduced hemolytic toxicity compared with the non-PEG modified dendrimers^17^,^18^.

Based on reports of cytotoxic effects of dendrimers, especially higher generation PAMAM (G4–G6) dendrimers, we investigated the hemolytic activity of G3 PAMAM dendrimers and its conjugates on erythrocytes isolated from adult male Sprague-Dawley rats^15^. The PEG_24_ modified G3 PAMAM dendrimers (700 μg/mL) and scFv6H4Cys (1300 μg/mL) concentration ranges used in the hemolysis studies were selected as these concentrations were used to synthesize the dendribodies. The dendribody concentration (400 μg/mL) used in the assay is the actual concentration measured using the bicinchoninic acid (BCA) assay after reacting 700 μg/mL PEG_24_ modified G3 PAMAM dendrimers with 1300 μg/mL scFv6H4Cys. The range of concentrations included the highest concentration of G3 PAMAM dendrimers that would be seen by tissues immediately after *in vivo* injection of the dendribodies. We found that native G3 PAMAM dendrimers were indeed hemolytic in a dose dependent manner, but were non-hemolytic after PEG modification or once converted to the dendribody format (Fig. 1b)^19^,^20^,^21^,^22^.

**Figure 3:**
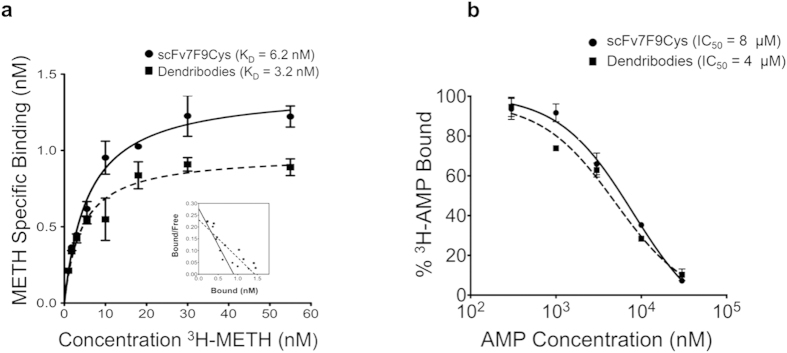
**a** *In vitro* saturation binding data for specific binding of [^3^H]-METH to scFv7F9Cys and dendribodies with corresponding Scatchard plots (inset). ScFv7F9Cys showed two-fold improvement in affinity for [^3^H]-METH after conjugation to the dendrimers (*p* < 0.05). The K_D_ values of the scFv7F9Cys and dendribodies were 6.2 and 3.2 nM respectively. **b** *In vitro* AMP competition binding data of percent [^3^H]-AMP bound to scFv7F9Cys and dendribodies. ScFv7F9Cys not only retained function but showed a two-fold increase in affinity for [^3^H]-AMP after conjugation to the dendrimers. The IC_50_ values of the scFv7F9Cys and dendribodies were 8 and 4 μM respectively. Data points are the mean ± SEM of triplicate determinations.

In order to determine if the scFv7F9Cys retained ligand-binding function upon dendrimer conjugation, *in vitro* functional saturation and competition binding assays were performed with METH and amphetamine (AMP), respectively. The affinities for [^3^H]-METH as measured by the dissociation constant (K_D_) were 6.2 and 3.2 nM for the scFv7F9Cys and dendribodies respectively (Fig. 3a). The AMP affinity (as measured by the IC_50_) were 8 and 4 μM for the scFv7F9Cys and dendribodies, respectively (Fig. 3b) The scFv7F9Cys based dendribodies showed statistically significant two-fold improvement in affinity for METH and AMP (*p* < 0.05) with respect to unconjugated scFv7F9Cys. These results indicate that conjugation of an anti-METH scFv to PEG modified dendrimer nanoparticle improved *in vitro* affinity to its ligand.

**Figure 4:**
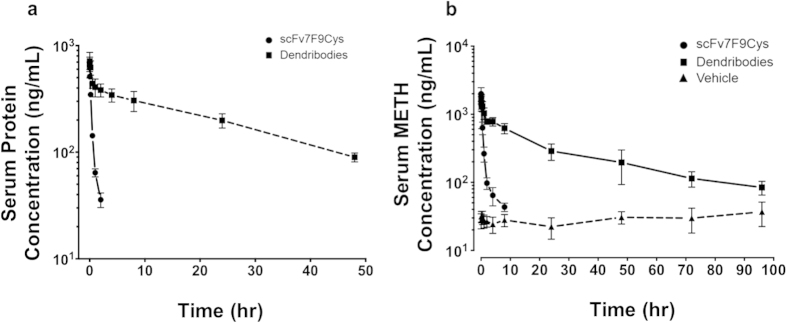
**a** Serum concentration-time profiles of anti-METH scFv7F9Cys and dendribodies after i.v. bolus dosing at t = 0 min. Average concentration-time profile of anti-METH scFv7F9Cys (closed circles, solid line) and dendribodies (closed squares, dashed line) after i.v. bolus dosing at t = 0 min. The total amount of antibody (μg/ml) in the serum was calculated based on the ratio of dose to radiolabeled protein dose (DPM). Connecting lines in the graph are representative and not the fitted curves used for PCKN modeling and determination of final PCKN parameters. **b** Average concentration versus time profiles for METH in serum with scFv7F9Cys (closed circles), dendribodies (closed squares) or with a control injection of vehicle (closed triangles) administered at t = 0 min. ScFv7F9Cys and dendribodies redistributed METH to the central compartment (vasculature) to cause an immediate increase in the METH concentrations. All groups of rats received an s.c. METH infusion of 3.2 mg/kg/day. All values are represented as the mean ± SEM; n = 6, scFv7F9Cys; n = 5 dendribody group.

To test the *in vivo* functionality and serum PCKN profiles of the dendribody we used an established rat model of chronic METH use. The average serum scFv7F9Cys and dendribody concentration-time profiles were biphasic curves that fit a two-compartment PCKN model (Fig. 4a). PCKN parameters of the unconjugated scFv7F9Cys and dendribodies were determined. Cls and volume of distribution (Vd) of scFv7F9Cys upon conjugation to dendrimer nanoparticle decreased 43 and 1.6 fold respectively, whereas the terminal elimination half-life (t_1/2λz_) increased 20 fold (Table 1). Based on the classic PCKN equation where t_1/2_ is a function of Cls and Vd, the drastic improvement in the t_1/2_ of scFv7F9Cys is primarily an effect of decreased Cls of scFv7F9Cys upon conjugation to dendrimers. The analysis of whole blood and serum at each time point revealed that scFv7F9Cys and dendribodies were limited to the serum compartment, with negligible protein association with the erythrocytes.

Antibody fragments are in general regarded as safe^5^. Anti-METH mAbs and prototype scFv6H4 in preclinical studies have shown no unfavorable effects up to 35 mg/kg^4^. However, since the dendribodies are an entirely new medication, all rats were observed frequently and regularly for signs of toxicity by monitoring general health, body weight, feeding behavior, hematocrit values, and by weighing and inspecting organs when animals were euthanized at the end of each study. All rats in the study maintained healthy body weight and no change in the food intake among the study groups was seen. Furthermore, macroscopic gross examination of the major organs revealed no anatomical localization or pathological changes. Thus, scFv7F9Cys and dendribodies appeared safe during the timeframe of these studies. Taken together, scFv7F9Cys and dendribodies had no observable toxic effects, agreeing with the *in vitro* hemolysis data and the safety studies published for the PEG modified dendrimers, a reaction intermediate that we used for conjugation.

*In vivo* tissue distribution studies were performed to determine the effect of change in the molecular mass of scFv7F9Cys in the dendribody format. We found that 7% of the injected dose per gram of tissue (ID/g) of the unconjugated scFv7F9Cys accumulated in the right kidney at 0.5 hr with negligible accumulation in the kidneys at 2 and 8 hr (Supplementary Fig. 1a and b). It has been previously reported that the scFvs (~25 KDa) are rapidly filtered through kidneys, since the molecular weight cut-off (MWCO) for glomerular filtration is ~60–70 KDa. During this process scFvs are often re-absorbed by the kidney tubules and metabolized^23^. For the dendribody group, some splenic accumulation (0.6% of injected dose) was observed at early time points (Supplementary Fig 1b), but no other significant differences in organ accumulation were observed.

In practice, it is generally considered that it takes 3 to 5 half-lives to completely remove a drug from the body, once the drug administration is discontinued. Thus t_1/2_ can be used as an indirect measurement to quantify elimination via Cls. Interestingly, representative radioactivity of 110% (24 hr) and 91% (144 hr) was detected in urine, in the form of [^3^H]-scFv7F9Cys and [^3^H]-dendribody, respectively (Supplementary Fig. 2). These data agree well with the estimated t_1/2λz_ of scFv7F9Cys (1.3 ± 0.3 hr) and dendribodies (26 ± 2.6 hr) in rats. Considering the molecular mass of dendribodies and the percent-injected radioactivity cleared in urine, it seems pertinent that the dendribodies could be catabolized *in vivo* to smaller units. For both study groups, the appearance of radioactivity in urine over time correlated well with its disappearance from blood suggesting that, for both therapeutics, renal clearance could be the major route of elimination. Overall, the blood, organ, and urine distribution of scFv7F9Cys and dendribodies correspond well with the PCKN data.

One measure of *in vivo* efficacy of the anti-METH mAbs is their ability to quickly and specifically bind METH and quickly redistribute it back to the vasculature. The average serum METH concentration over time curves showed that both scFv7F9Cys and dendribodies redistributed METH to the vascular compartment to cause an immediate 61 and 63 fold increase in the METH concentrations respectively compared to the control group (Fig. 4b). However the dendribodies maintained higher serum METH concentrations for 96 hr compared to 8 hr by scFv7F9Cys (Fig. 4b).

It has been previously shown that the anti-METH mAb-bound METH tends to adopt the PCKN profile of the mAb itself and thus the t_1/2_ of METH appeared to increase in the dendribody treated rats. When METH concentrations in the presence of scFv7F9Cys and dendribodies were plotted as their respective molar concentrations, the relationship was nearly 1:2 (Supplementary Fig. 3). This could be due to a certain population of scFv7F9Cys and dendribody administered that could have been inactive or inactivated *in vivo* over time. Nevertheless, we noticed that the serum METH area under the concentration versus time curve (AUC_0-last_) for dendribodies increased 15-fold compared to scFv7F9Cys treated rats. Thus suggesting that the Cls of METH was decreased in the presence of dendribodies compared with scFv7F9Cys treated rats. Overall the dendribody format prolonged the ability of scFv7F9Cys to bind METH *in vivo* over a period of 96 hr. We anticipate that this improved efficacy could result in some neuroprotective effect against METH induced adverse effects in further studies. In conclusion, this proof-of-concept study provides *in vivo* evidence that PCKN properties and efficacy of an anti-METH scFv can be improved by creatively combining molecular engineering and nanotechnology. Additionally, this dendribody platform capable of generating multivalent medications with customizable PCKN profiles could be adapted to increase the serum residence time for other biological therapies for various diseases and organ-specific targeting.

## Methods

### Chemicals and drugs

Enzymes and *Escherichia coli* strains were purchased from Invitrogen (Carlsbad, CA). [^3^H]-N-Methyl-1-phenylpropane-2-amine (METH) (39 Ci/mmol) labeled at two metabolically stable sites on the aromatic ring structure was obtained from the National Institute on Drug Abuse (Bethesda, MD) after synthesis at the Research Triangle Institute (Research Triangle Park, NC). G3 PAMAM dendrimers were purchased from Dendritic Nanotechnologies, Inc. (Mt. Pleasant, MI). The heterobifunctional cross-linker with N-hydroxysuccinimide (NHS) ester and maleimide groups with a 24 PEG group spacer (PEG_24_) was purchased from Thermo. (±)-METH and (±)-1-phenyl-1,2-dideutero-2-[trideuteromethyl]aminopropane [(±)-METH-D5] used for mass spectroscopy were obtained from Cerilliant (Round Rock, TX). Optima LC-MS grade methanol, formic acid, acetonitrile, glacial acetic acid, isopropanol, ammonium hydroxide and all other reagents were procured from Thermo Fisher Scientific (Pittsburgh, PA) or Sigma Aldrich (St. Louis, MO) unless otherwise noted. All drug doses were calculated as the free base form.

### *In vitro* hemolysis assay

Erythrocytes isolated from the blood of adult male Sprague Dawley rats were suspended in phosphate buffered saline (PBS buffer: 2.68 mM KCl, 1.47 mM KH_2_PO_4_, 136.89 mM NaCl, 8.10 mM Na_2_HPO_4_, pH 7.3), or PBS buffer containing G3 PAMAM dendrimers (concentrations ranging from 5 to 1000 μg/ml), PEG_24_ modified G3 PAMAM dendrimers (700 μg/ml), scFv6H4Cys (1300 μg/ml), and dendribodies (400 μg/ml). These suspensions were placed in an incubator shaker at 37 °C for 4 hr. The erythrocyte suspensions were then centrifuged at 1000 *g* for 5 min. The percent hemolysis was determined on the basis of released hemoglobin in the supernatants measured spectrophotometrically at 540 nm. As a positive control erythrocytes were treated with distilled water and PBS was used as a negative control. The absorbance value obtained for the supernatant of the erythrocytes treated with distilled water was taken to indicate 100% hemolysis whereas PBS was considered 0% hemolytic.

### ScFv7F9Cys design, production, and purification

General molecular and genetic techniques for cDNA cloning and sequencing of scFv7F9Cys were performed as described in Sambrook and Russell, 2001^24^. A carboxyl terminus cysteine was engineered immediately following the 6His tag of scFv7F9 by DNA synthesis to create scFv7F9Cys. Catalent Pharma Solutions (Somerset, NJ) was contracted to transfect a Chinese hamster ovary (CHO) cell line for producing the recombinant scFv7F9Cys. Catalent utilized a proprietary transfection technology for introducing scFv7F9Cys cDNA into the CHO cells. ScFv7F9Cys CHO cells were stored in a liquid nitrogen cryo-freezer until thawed for cell culture. Under growth conditions optimized by Catalent Pharma Solutions, scFv7F9Cys was produced in gram quantities with cell culture using a Wave Bioreactor System (GE Healthcare, Piscataway, NJ). Briefly, under sterile conditions, scFv7F9Cys CHO cells were removed from liquid nitrogen storage, rapidly thawed in a 37 °C water bath, suspended in growth media, centrifuged at 250 *g* for 5 min, and the supernatant was decanted. The pelleted CHO cells were re-suspended in culture media and transferred to a 25 cm^2^ static culture flask in a CO_2_ incubator with 5% CO_2_ and 95% humidity. Cell count and viability were monitored daily by staining small aliquots with trypan blue to detect lysed or apoptotic cells. Static cultures were expanded to 225 cm^2^ flasks and cultured to a cell density of approximately 1 × 10^6^ cells/ml with viability above 97%, reaching the exponential growth phase in 8 to 10 days, at which time 20 L Wave bioreactor bags were inoculated with 1.2 L CHO cell culture and 2 L fresh culture media to reach a starting cell count of >3 × 10^5^ cells/ml. The Wave bioreactor bags were incubated for 7 to 8 days with daily monitoring of cell counts, pH, ratios of partial pressure of carbon dioxide to partial pressure of oxygen (pCO_2_/pO_2_), glucose, and lactate levels using a Bayer blood gas analyzer. Supplemental Cellboost media containing 3 mM glutamine was added on days 2, 4, and 6 to a maximum of 10 L total volume. Cells were harvested by centrifugation at 2800 *g* for 10 min when viability dropped below 50% and total living cell count was <3 × 10^5^ cells/ml. Culture supernatant containing secreted scFv7F9Cys was stored at −80 °C until purification and formulation. To harvest and analyze total scFv7F9Cys expression, the samples were centrifuged at 20,000 *g* for 10 min, and the supernatant was analyzed using SDS−PAGE and Coomassie dye G-250 based GelCode Blue staining. The scFv7F9Cys expression product was purified using an AKTA explorer 100 FPLC system and a nickel sepharose IMAC HiPrep FF16/10 column (GE Healthcare, Piscataway, NJ)^4^.

### Synthesis, scale-up, and purification of scFv7F9Cys based dendribodies

PEG_24_ modified G3 PAMAM dendrimers were synthesized, characterized and further reacted with DTT reduced scFv7F9Cys to produce dendribodies as previously described^6^. The dendribody reaction was scaled up in endotoxin-free plastic and glassware. The factors influencing the scFv6H4Cys based dendribody production were screened and optimized for a reaction volume of 0.07 ml. The production of scFv7F9Cys based dendribodies was scaled up to a 15 ml conjugation reaction. The dendribody reaction was scaled in eleven batches of 30 mg scFv7F9Cys per 15 ml batch. ScFv7F9Cys based dendribodies were purified by SEC using an AKTA Explorer 100 FPLC and a 350 ml Superdex 200/16 column (GE Healthcare, Piscataway, NJ). In brief, the column was equilibrated with 5 column volumes of molecular weight sizing buffer (20 mM sodium phosphate buffer, pH 7.4, 500 mM NaCl) at 2.5 ml/min. The dendribody reaction mixture containing the unreacted PEG_24_ modified dendrimers and scFv7F9Cys, unconjugated scFv7F9Cys, and dendribodies was loaded onto the column. The dendribodies were eluted from the column and collected as 0.5 ml fractions. The elution profile was monitored by UV absorbance at 220, 254, and 280 nm wavelengths. All fractions were analyzed by SDS−PAGE, and the dendribody-enriched fractions were pooled and concentrated. ScFv7F9cys and dendribody concentrations were estimated using micro BCA assay (Pierce Thermo Scientific, Waltham, MA) as per manufacturer’s instructions.

### Saturation and competition binding assays for determination of equilibrium dissociation constants (K_D_) and half minimal inhibitory concentration (IC_50_) values of scFv7F9Cys and scFv7F9Cys based dendribodies for METH and AMP respectively

The binding affinities (K_D_s) of scFv7F9Cys and dendribodies for METH were determined using a saturation binding assay as previously described for scFv6H4Cys and scFv6H4Cys based dendribodies^4^,^6^. The AMP binding of scFv7F9Cys and scFv7F9Cys based dendribodies were evaluated using competition binding assays measuring IC_50_ value as previously described^25^.

### Tritium labeling of scFv7F9Cys and scFv7F9Cys based dendribodies

ScFv7F9Cys and dendribodies were labeled with N-Succinimidyl [2,3-^3^H] Propionate ([^3^H]-NSP) for the serum PCKN studies as described in McClurkan *et al.*^26^,^27^. The resulting [^3^H]-scFv7F9Cys and [^3^H]-dendribodies were used as a tracer dose to allow accurate determination of scFv7F9Cys and dendribody concentrations over time in the rats. Briefly, 500 μCi of [^3^H]-NSP was dried to remove solvents under a light stream of argon gas in a siliconized tube. The scFv7F9Cys or dendribodies (0.5–1 mg/500 μl in PBS, pH 7.4) was added to the tube and then this solution was placed in the reaction vial along with a stir bar. The reaction was stirred for 4 hr on ice in the dark. The reaction mixture was then passed over a PD10 G-25 gel filtration column (GE Healthcare) that had been equilibrated with PBS to separate unincorporated [^3^H]-NSP from [^3^H]-protein. Fractions containing [^3^H]-scFv7F9Cys or [^3^H]-dendribodies that were detected using absorbance at 280 nm were combined and dialyzed against PBS using 3 kDa MWCO Slide-A-Lysers (Thermo Fisher Scientific). These dialyzed [^3^H]-scFv7F9Cys or [^3^H]-dendribodies were further analyzed using SEC to confirm tritium labeling.

### Pharmacokinetic studies of scFv7F9Cys, scFv7F9Cys based dendribodies, and METH in rats

All methods were done in accordance with and approved by The Institutional Animal Care and Use Committee at University of Arkansas for Medical Sciences (UAMS). Detailed PCKN studies were designed with the following groups: control (vehicle), scFv7F9Cys, and dendribodies using dual jugular vein catheterized adult male Sprague-Dawley rats (n = 6 per group) purchased from Charles River Laboratories (Raleigh, NC). Animals were housed individually in a light-controlled environment (12 hr light/dark cycle). They received water *ad libitum* and were fed approximately 30 g of food pellets daily, which maintained their body weights between 280–310 g. All experiments were conducted with the prior approval of the Institutional Animal Care and Use Committee of UAMS. On day -4 (i.e., 4 days prior to dosing) of the study, pre-METH blood samples (150 μl) were taken and on day -3, the rats received 3.2 mg/kg/day METH via subcutaneous Alzet 15 day osmotic pumps (Durect Corp, Cupertino, CA). On day -1, blood samples (150 μl) were taken at 10 am, 1, and 5 pm to allow determination of METH control steady-state concentrations and the rats were placed in metabolic cages for urine and feces collection. On Day 0, the rats were given an i.v. bolus dose of anti-METH protein formulation of 36.5 mg/kg containing a tracer dose (~100 μg) of 5 × 10^6^ DPM [^3^H]-protein or an equal volume of vehicle (formulation buffer without protein and tracer dose). This 36.5 mg/kg dose was calculated to be equimolar to the METH body burden at steady state. Immediately following administration of vehicle, scFv7F9Cys, or dendribodies, blood samples (150 μl) were taken at various time points (1, 5, 10, 30 min; 1, 2, 4, 8, 24, 48, 72, 96, 120, 144, and 168 hr) until radioactivity detected in these samples was near background levels. A small aliquot of whole blood was used to determine the hematocrit values (the proportion of the blood volume occupied by erythrocytes) in heparinized tubes using standard procedures every day of the study. As indicators of medication safety, rats were continuously monitored for general health, hematocrit values, body weight, feeding behavior and at the end of the study after euthanasia, their major organs were weighed and visually inspected for signs of toxicity.

ScFv7F9Cys and dendribody blood to serum ratios were determined using blood samples collected during the PCKN studies. Duplicate whole blood samples (5 μl) were added to 5 ml liquid scintillation fluid (Ecoscint A, National Diagnostics, Atlanta, GA) for quantification by liquid scintillation spectrophotometry. The remaining blood was allowed to clot, centrifuged, and the serum fraction was collected. Duplicate 5 μl aliquots of the serum were used to determine the [^3^H]-scFv7F9Cys and [^3^H]-dendribody concentration in serum, as described for the whole blood. The ratio of the concentration of scFv7F9Cys or dendribodies in the whole blood to that in the serum was used to represent the distribution of scFv7F9Cys and dendribodies between erythrocytes and serum. At the end of the study, rats were anesthetized with isoflurane (Forane^®^, Baxter Heathcare Corporation, Deerfield, IL) and sacrificed by decapitation. Major organs (brain, heart, liver, kidney, spleen, pancreas and testes) were immediately collected, weighed and stored at −80 °C along with serum samples until analysis. To determine the molar concentration of unlabeled scFv7F9Cys and dendribodies in the blood, the known amounts of [^3^H]-scFv7F9Cys and [^3^H]-dendribody radioactivity in the blood were converted to the relative molar concentration of scFv7F9Cys and dendribodies, respectively. The calculations were based on a 5 × 10^6^ DPM [^3^H]-protein dose and a 36.5 mg/kg dose of unlabeled protein per rat. Based on the sequence of scFv7F9Cys, a theoretical molecular weight of 27 kDa was calculated using the ExPASy Compute pI/Mw web tool^28^.

### Estimation of serum METH concentrations using liquid chromatography with tandem mass spectrometry (LC-MS/MS) analysis

For analysis of METH serum concentrations, Strata X-C 33 μM Polymeric Strong Cation solid phase extraction columns (Phenomenex, Torrance, CA) were conditioned with 2 ml methanol and 2 ml loading buffer (100 mM NaPO_4_, pH 8.1). A METH standard curve in Sprague Dawley rat non-hemolyzed serum (Pel-Freez Biologics, Rogers, AR) with concentrations ranging from 0.3–2000 ng/ml and quality control standards were prepared for analysis of serum as previously described^29^. For each sample or standard, 25 μl aliquots were mixed with 25 μl of internal standard (100 ng/ml METH-D5) and allowed to equilibrate to precipitate proteins before 1 ml of loading buffer (50 mM sodium phosphate buffer, pH 6.7, 0.1 M sodium sulfate) was added. The mixture was then transferred to the solid phase extraction column and rinsed with methanol. After drying the column under vacuum, elution buffer [5% (v/v) ammonium hydroxide, 5% (v/v) tetrahydrofuran, and 90% (v/v) acetonitrile] was used to elute the METH into glass tubes containing 0.5 M hydrochloric acid. The samples were then dried under nitrogen gas, reconstituted with 0.1% (v/v) formic acid, and centrifuged to remove any potential precipitates.

The concentrations of METH were then determined using an Acquity Ultra Performance Liquid Chromatography system interfaced with a Quattro Premier XE mass spectrometer (Waters Corp, Milford, MA). The analytes were separated using a linear binary gradient with reversed phase chromatography at a flow rate of 0.3 ml/min [mobile phase A: 0.1% (v/v) formic acid in water; mobile phase B: 0.1% (v/v) formic acid in acetonitrile]. The gradient program was as follows: 0–1 min: 10% B; 1–4 min: 10%–90% B and held for 1 min; 5–6 min: 90%–10% B. The total run time was 7 min. Each sample (5 μl) was injected onto an Acquity UPLC BEH C18 1.7 μm [2.1 (i.d.) × 100 mm] column (Waters Corp). The column was maintained at 40 °C and coupled to an electrospray ionization probe, which was operated in the positive ion mode. Positive ions for METH and METH-D5 were generated at cone voltages of 18 and 21 volts, respectively. Product ions METH and METH-D5 were generated using argon collision induced disassociation at collision energy of 15 and 21 eV while maintaining a collision cell pressure of 3 × 10^−3^ torr. Detection was achieved in the multiple-reaction-monitoring (MRM) mode using the precursor → product ions, *m/z* 149.8 → 90.8 and 155.2 → 91.5 for METH and METH-D5, respectively. The lower and upper limit of quantification for METH was 1 and 2000 ng/ml, respectively. All predicted values for standards were within ±20% (Supplementary Fig. S4).

### Biodistribution studies of scFv7F9Cys and scFv7F9Cys based dendribodies

At 30 min, 2, and 4 hr post i.v. injection of [^3^H]-scFv7F9Cys (3.5 × 10^6^ DPM) and 2, 4, and 8 hr post i.v. injection of [^3^H]-dendribodies (3.5 × 10^6^ DPM) adult male Sprague Dawley rats (n = 2 per time point) were euthanized and major organs (lung, heart, liver, kidney, spleen, pancreas, testis, and brain) were harvested and weighed. The organs were chemically solubilized with Solvable™ solution (Packard, Downers Grove, IL, USA) as per manufacturer’s instructions and samples incubated overnight at 50–55 °C. The samples were subsequently decolorized with 200–300 μL of 30% (v/v) hydrogen peroxide (Sigma-Aldrich, St. Louis, MO) to avoid color quenching of the scintillation cocktail. The amount of tritium per sample was determined via liquid scintillation counting using 10 ml liquid scintillation fluid (Ecoscint A, National Diagnostics, Atlanta, GA) per sample. The results were then expressed as a percentage of the injected dose per gram of tissue (% ID/g) without residual blood correction. Thus, the resultant organ biodistribution data are directly comparable between rats in different study groups.

### Data and statistical analysis

The saturation and competition binding data were analyzed by non-linear curve fitting using Graphpad PRISM^®^ 5.0d software. Affinity (K_D_) for METH saturation binding was determined using nonlinear curve fitting that accounted for specific and nonspecific binding. IC_50_ values for AMP binding to scFv7F9Cys and dendribodies were determined from the percent [^3^H]-AMP bound versus unlabeled AMP concentration curves^30^. Unpaired, one-tailed Student’s t-test was performed to obtain the statistical significance of the differences in affinity values in K_D_ (METH) and IC_50_ (AMP) for scFv7F9Cys and dendribodies determined in buffer. Data represent mean ± standard error of the mean (SEM) for the number (n) of experiments indicated in figure legends. A significance level of p < 0.05 was used for all studies. All PCKN analyses were performed using WinNonlin version 5.0 (Pharsight Corporation, Mountain View, CA). Serum PCKN parameters were derived from model-dependent analysis of the average concentration versus time curves for scFv7F9Cys and dendribodies. A curve was fit to the data points using a two-compartment i.v. bolus model, with no weighting, 1/y, or 1/y^2^ weighting. The best-fit line was chosen by visual inspection and analysis of the residuals. The PCKN calculations included *t*_1/2λz_, Cls, and Vd at steady-state. To assess the effect of scFv7F9Cys and dendribodies on METH serum concentrations at each time point, a multiple comparison was performed using two-way analysis of variance (ANOVA) for three groups and Student’s t-test for two groups. Statistical differences were set at the p < 0.05 levels. All tests were performed using Graphpad PRISM^®^ 6.0d software.

## Additional Information

**How to cite this article**: Nanaware-Kharade, N. *et al.* A Nanotechnology-Based Platform for Extending the Pharmacokinetic and Binding Properties of Anti-methamphetamine Antibody Fragments. *Sci. Rep.* **5**, 12060; doi: 10.1038/srep12060 (2015).

## Supplementary Material

Supplementary Information

## Figures and Tables

**Table 1:** Average pharmacokinetic values determined from rat serum concentrations for scFv7F9Cys and dendribodies in the presence of steady state METH infusions.

**PCKN Parameter ± SEM**	**scFv7F9Cys**	**Dendribodies**	**Fold Change**
t_1/2λz_ (hr)	1.3 ± 0.3	26.6 ± 2.6	⬆20
Cls (ml/min/kg)	2 ± 0.04	0.044 ± 0.0015	⬇45
Vd (ml/kg)	152 ± 10.7	98 ± 2.9	⬇1.6

All values are represented as the mean ± SEM; n = 6, scFv7F9Cys; n = 5 dendribody group.

## References

[b1] Laurenzana E. M. *et al.* Use of anti-(+)-methamphetamine monoclonal antibody to significantly alter (+)-methamphetamine and (+)-amphetamine disposition in rats. Drug Metab Dispos 31, 1320–1326 (2003). 14570763 10.1124/dmd.31.11.1320

[b2] Byrnes-Blake K. *et al.* Pharmacodynamic mechanisms of monoclonal antibody-based antagonism of (+)-methamphetamine in rats. Eur J Pharmacol 461, 119–128 (2003). 12586207 10.1016/s0014-2999(03)01313-x

[b3] Owens S. M., Atchley W. T., Hambuchen M. D., Peterson E. C. & Gentry W. B. Monoclonal antibodies as pharmacokinetic antagonists for the treatment of (+)-methamphetamine addiction. CNS Neurol Disord-DR 10, 892–898 (2011). 10.2174/187152711799219370 PMC3653579 22229314

[b4] Peterson E. C., Laurenzana E. M., Atchley W. T., Hendrickson Howard P. & Owens S. M. Development and preclinical testing of a high-affinity single-chain antibody against (+)-methamphetamine. J Pharmacol Exp Ther 325, 124–133 (2008). 18192498 10.1124/jpet.107.134395 PMC2773181

[b5] Minckwitz, von G. *et al.* Phase I clinical study of the recombinant antibody toxin scFv(FRP5)-ETA specific for the ErbB2/HER2 receptor in patients with advanced solid malignomas. Breast Cancer Res. 7, R617–26 (2005). 16168106 10.1186/bcr1264 PMC1242130

[b6] Nanaware-Kharade N., Gonzalez G. A. III, Lay J. O. Jr, Hendrickson Howard P. & Peterson E. C. Therapeutic Anti-Methamphetamine Antibody Fragment-Nanoparticle Conjugates: Synthesis and *in Vitro* Characterization. Bioconjug Chem 23, 1864–1872 (2012). 22873701 10.1021/bc300204n PMC3463141

[b7] Holliger P. & Hudson P. Engineered antibody fragments and the rise of single domains. Nat Biotechnol 23, 1126–1136 (2005). 16151406 10.1038/nbt1142

[b8] Constantinou A., Chen C. & Deonarain M. P. Modulating the pharmacokinetics of therapeutic antibodies. Biotechnol Lett 32, 609–622 (2010). 20131077 10.1007/s10529-010-0214-z

[b9] Dolezal O. *et al.* ScFv multimers of the anti-neuraminidase antibody NC10: shortening of the linker in single-chain Fv fragment assembled in V(L) to V(H) orientation drives the formation of dimers, trimers, tetramers and higher molecular mass multimers. Protein Eng 13, 565–574 (2000). 10964986 10.1093/protein/13.8.565

[b10] Yang K. *et al.* Tailoring structure-function and pharmacokinetic properties of single-chain Fv proteins by site-specific PEGylation. Protein Eng 16, 761–770 (2003). 14600206 10.1093/protein/gzg093

[b11] Kubetzko S., Sarkar C. A. & Plückthun A. Protein PEGylation decreases observed target association rates via a dual blocking mechanism. Mol Pharmacol 68, 1439–1454 (2005). 16099846 10.1124/mol.105.014910

[b12] Kubetzko S., Balic E., Waibel R., Zangemeister-Wittke U. & Pluckthun A. PEGylation and Multimerization of the Anti-p185HER-2 Single Chain Fv Fragment 4D5: Effects on Tumor Targeting. J Biol Chem 281, 35186–35201 (2006). 16963450 10.1074/jbc.M604127200

[b13] Ahmad K. M., Xiao Y. & Soh H. T. Selection is more intelligent than design: improving the affinity of a bivalent ligand through directed evolution. Nucleic Acids Res 40, 11777–11783 (2012). 23042245 10.1093/nar/gks899 PMC3526301

[b14] Stevens M. W. *et al.* Preclinical characterization of an anti-methamphetamine monoclonal antibody for human use. MAbs 6, 547–555 (2014). 24492290 10.4161/mabs.27620 PMC3984342

[b15] Domański D. M., Klajnert B. & Bryszewska M. Influence of PAMAM dendrimers on human red blood cells. Bioelectrochemistry 63, 189–191 (2004). 15110271 10.1016/j.bioelechem.2003.09.023

[b16] Hong S. *et al.* Interaction of poly(amidoamine) dendrimers with supported lipid bilayers and cells: hole formation and the relation to transport. Bioconjug Chem 15, 774–782 (2004). 15264864 10.1021/bc049962b

[b17] Kolhe P., Misra E., Kannan R. M., Kannan S. & Lieh-Lai M. Drug complexation, *in vitro* release and cellular entry of dendrimers and hyperbranched polymers. Int J Pharm 259, 143–160 (2003). 12787643 10.1016/s0378-5173(03)00225-4

[b18] Qi R. *et al.* PEG-conjugated PAMAM dendrimers mediate efficient intramuscular gene expression. AAPS J 11, 395–405 (2009). 19479387 10.1208/s12248-009-9116-1 PMC2758109

[b19] Kameneva M. V., Repko B. M., Krasik E. F., Perricelli B. C. & Borovetz H. S. Polyethylene glycol additives reduce hemolysis in red blood cell suspensions exposed to mechanical stress. ASAIO J. 49, 537–542 (2003). 14524560 10.1097/01.mat.0000084176.30221.cf

[b20] Klajnert B., Pikala S. & Bryszewska M. Haemolytic activity of polyamidoamine dendrimers and the protective role of human serum albumin. P Roy Soc A-Math Phy 466, 1527–1534 (2010).

[b21] Malik N. *et al.* Dendrimers: relationship between structure and biocompatibility *in vitro*, and preliminary studies on the biodistribution of 125I-labelled polyamidoamine dendrimers *in vivo*. J Control Release 65, 133–148 (2000). 10699277 10.1016/s0168-3659(99)00246-1

[b22] Roberts J. C., Bhalgat M. K. & Zera R. T. Preliminary biological evaluation of polyamidoamine (PAMAM) Starburst dendrimers. J Biomed Mater Res 30, 53–65 (1996). 8788106 10.1002/(SICI)1097-4636(199601)30:1<53::AID-JBM8>3.0.CO;2-Q

[b23] Arano Y. Strategies to reduce renal radioactivity levels of antibody fragments. Q J Nucl Med 42, 262–270 (1998). 9973841

[b24] Sambrook J. & Russell D. W. Molecular Cloning. (CSHL Press, 2001).

[b25] Thakkar S., Nanaware-Kharade N., Celikel R., Peterson E. C. & Varughese K. I. Affinity improvement of a therapeutic antibody to methamphetamine and amphetamine through structure-based antibody engineering. Sci Rep 4, 3673 (2014). 24419156 10.1038/srep03673 PMC4070344

[b26] McClurkan M., Valentine J., Arnold L. & Owens S. Disposition of a monoclonal anti-phencyclidine Fab fragment of immunoglobulin G in rats. J Pharmacol Exp Ther 266, 1439–1445 (1993). 8371148

[b27] Müller G. H. Protein labelling with 3H-NSP (N-succinimidyl-[2,3-3H]propionate). J Cell Sci 43, 319–328 (1980). 7419623 10.1242/jcs.43.1.319

[b28] Gasteiger E. *et al.* ExPASy: The proteomics server for in-depth protein knowledge and analysis. Nucleic Acids Res 31, 3784–3788 (2003). 12824418 10.1093/nar/gkg563 PMC168970

[b29] Hendrickson H., Laurenzana E. & Owens S. M. Quantitative determination of total methamphetamine and active metabolites in rat tissue by liquid chromatography with tandem mass spectrometric detection. AAPS J 8, E709–17 (2006). 17233534 10.1208/aapsj080480 PMC2751367

[b30] Akera T. & Cheng V. A simple method for the determination of affinity and binding site concentration in receptor binding studies. Biochim Biophys Acta 470, 412–423 (1977). 144525 10.1016/0005-2736(77)90132-8

