# Supplementary material for: A Nanotechnology-Based Platform for Extending the Pharmacokinetic and Binding Properties of Anti-methamphetamine Antibody Fragments
Source: Sci Rep. 2015 Jul 10;5:12060. doi: 10.1038/srep12060 (PMC4498229; doi:10.1038/srep12060)
Supplement: Supplementary Information [file srep12060-s1.pdf]

A Nanotechnology-Based Platform for Extending the Pharmacokinetic and Binding Properties of Anti-methamphetamine Antibody Fragments

Nisha Nanaware-Kharade[1†], Shraddha Thakkar[2], Guillermo A. Gonzalez III[1‡], and Eric C. Peterson[1*]

[1]Department of Pharmacology and Toxicology, College of Medicine, University of Arkansas for Medical Sciences, 4301 West Markham St., # 611, Little Rock, Arkansas 72205, USA.

[2]Department of Physiology and Biophysics, College of Medicine, University of Arkansas for Medical Sciences, 4301 West Markham St., # 750, Little Rock, Arkansas 72205, USA.

*email: EPeterson@uams.edu

Supplementary Figures

Figure S1

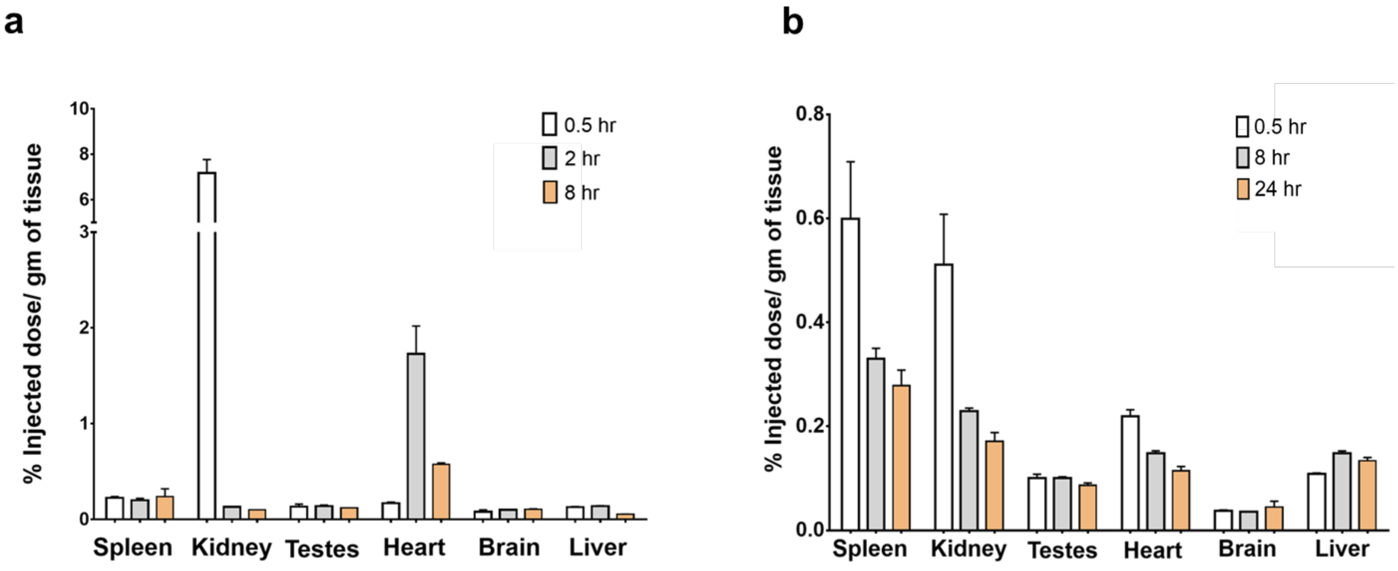

Supplementary Figure S1 | a, Organ distribution of scFv7F9Cys in male Sprague Dawley rats at three time points: 0.5, 2, and 8 hr post-injection depicted as percent injected dose recovered per gram of organ (%ID/g). **b**, Organ distribution of dendribodies in male Sprague Dawley rats at three time points: 0.5, 2, and 24 hr post-injection depicted as percent injected dose recovered per gram of organ (%ID/g). All values are represented as the mean ± range; n = 2 per time point. Note different Y axes in panels a and b.

Figure S2

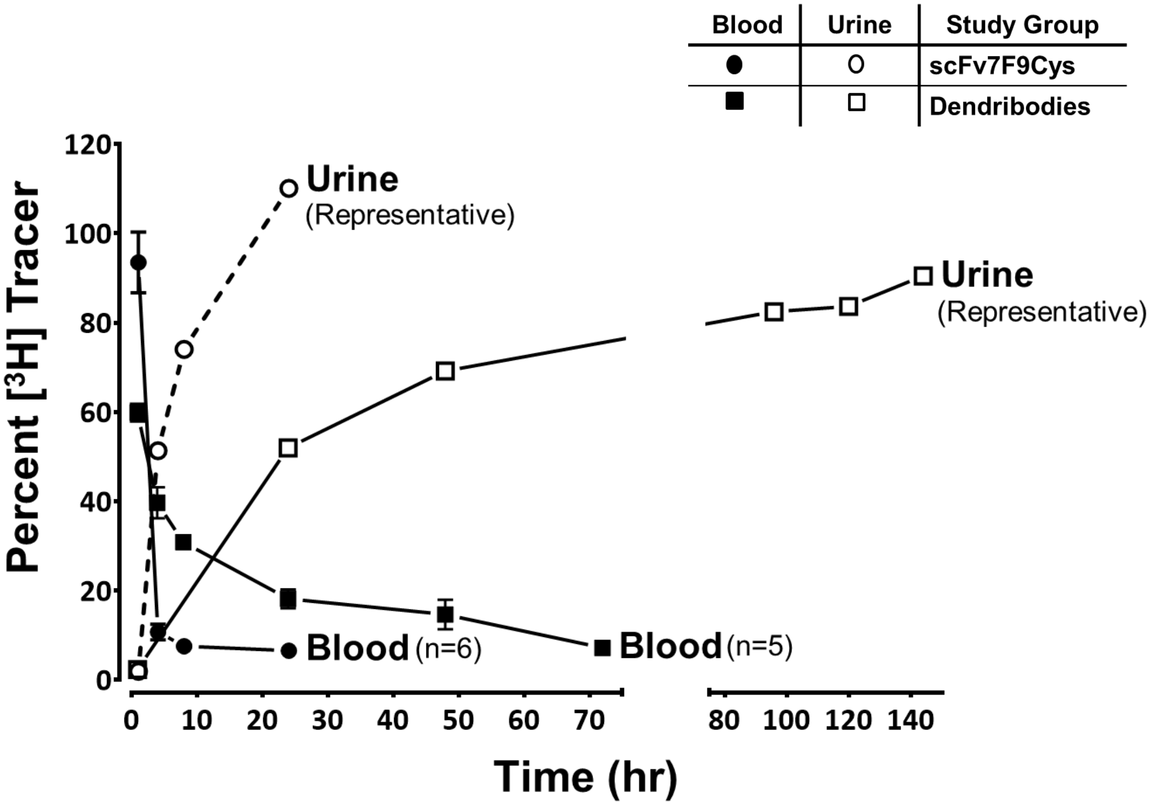

Supplementary S2 | Whole blood and urine profile of [^{3}H]-scFv7F9Cys and [^{3}H]-dendribody tracer dose shown as cumulative dose in blood and urine over time. Representative radioactivity of 110% (1440 min) and 91% (8640 min) was detected in urine for [^{3}H]-scFv7F9Cys and [^{3}H]-dendribody, respectively. These data concur with the estimated $t_{1/2\lambda z}$ of scFv7F9Cys (1.3 ± 0.3 hr) and dendribodies (26 ± 2.6 hr). Renal clearance appears to be the major route of elimination for both the groups. All values are represented as the mean ± SEM; n = 6 for scFv7F9Cys and n = 5 for dendribody blood circulation profiles along with a representative urine profile for both the groups.

Figure S3

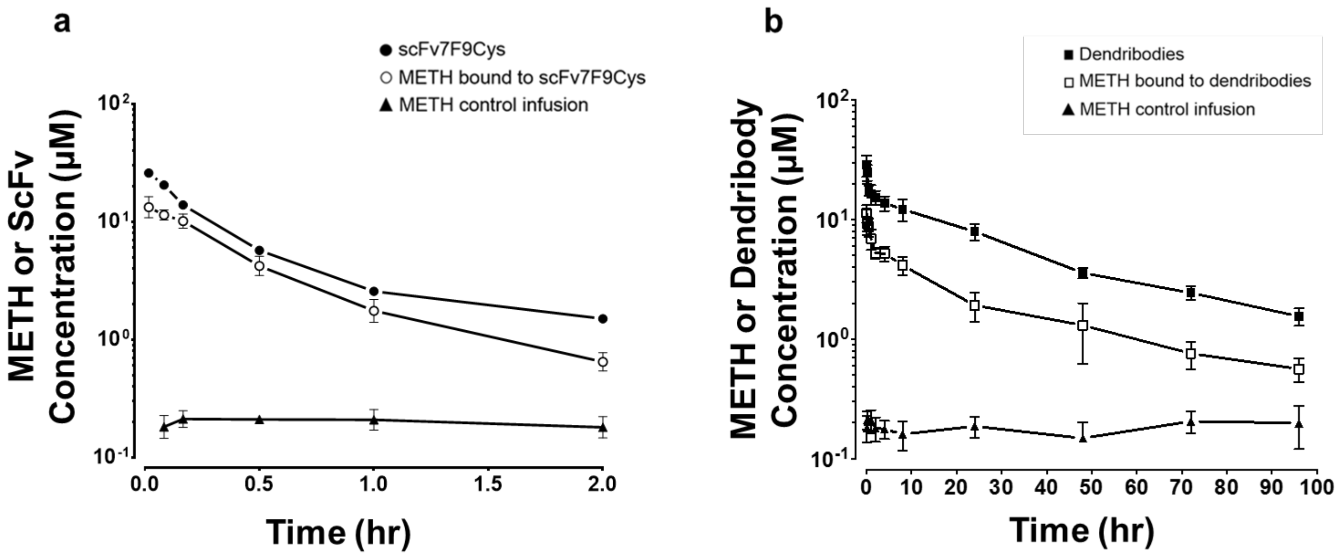

Supplementary S3 | a, Molar concentrations of METH (open circles), in the presence of scFv7F9Cys (closed circles), and METH without scFv (closed triangles). All concentration values are shown as µM concentrations versus time. All values are represented as the mean ± SEM; n = 6. **b**, Molar concentrations of METH (open squares), in the presence of dendribodies (closed squares), and METH steady-state concentration without dendribody (closed triangles). All concentration values are shown as µM concentrations versus time. All values are represented as the mean ± SEM; n = 5.

Figure S4

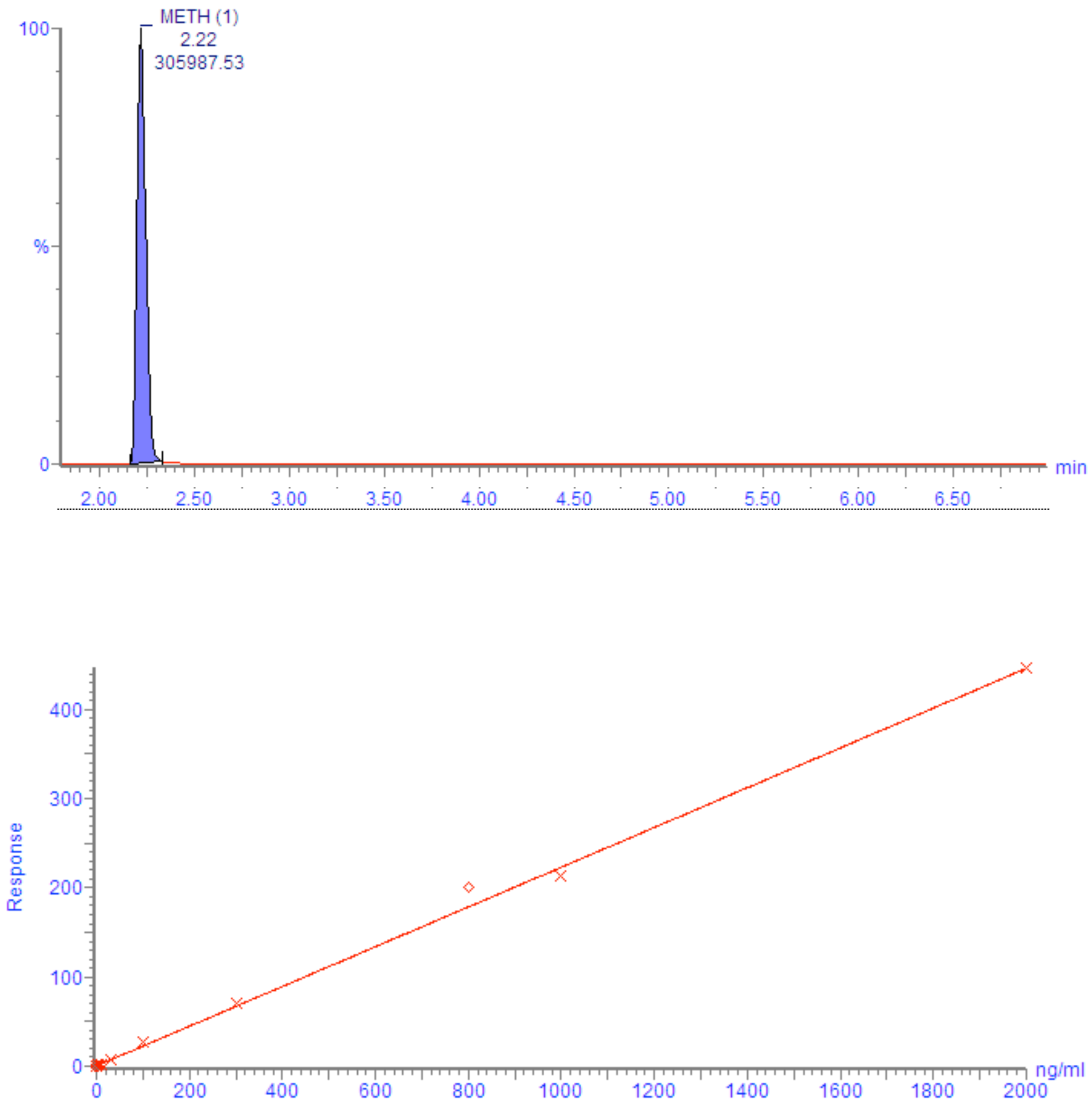

Supplementary S4 | Upper panel, Representative LC-MS/MS chromatogram showing extracted serum containing 300 ng/ml METH. **Lower panel**, Representative LC-MS/MS standard curve of METH from 1 to 2000 ng/ml. All predicted values for standards were within ± 20%
